# What are patients saying about their experience with atopic dermatitis? Insights from a machine learning analysis of online comments

**DOI:** 10.1002/ski2.100

**Published:** 2022-02-02

**Authors:** Y. Yin, K. Capozza, Y. Shao, M. Tu, Phillip Ma, Q. Zeng‐Treitler, A. A. Sun, I. A. Myles

**Affiliations:** ^1^ George Washington University Washington District of Columbia USA; ^2^ Global Parents for Eczema Research Santa Barbara California USA; ^3^ Epithelial Therapeutics Unit National Institute of Allergy and Infectious Disease National Institutes of Health Bethesda Maryland USA

## ETHICS STATEMENT

The project was deemed IRB‐exempt by the IRB of NIH.

Dear Editor,

1

Atopic dermatitis (AD) is a chronic skin condition and the most common form of eczema. It affects approximately 5%–34% of children and 1%–9% of adults in industrialised nations^1^ and generates a wide range of negative psychosocial and quality‐of‐life impacts for patients and their families.^2^ However, the pace of clinical practice can limit a providers' ability to address the full spectrum of issues patients with AD (and their caregivers) face. Comments shared in online patient communities may therefore provide missing insights into the burdens and unmet needs in the AD patient community.

We extracted 264 747 anonymous posts from publicly available online patient communities posted between November 2009 and January 2021 and used machine learning techniques, particularly Natural Language Processing (NLP), to classify posts and filter out irrelevant comments such as advertisements or greetings. Topic modelling was then performed in three successive rounds on 199 537 total relevant posts with MALLET Latent Dirichlet allocation. The analysis generated 344 stable topics which were grouped into categories with input from patients, caregivers, and bioinformaticians. Topics were seen as representative of the post if the topic weight was greater than 0.01.

The most discussed content in the sample of nearly 200 000 posts related to the psychosocial impacts of eczema (Table 1). Comments included discussions of feelings of sadness and anger; description of the social isolation and stigmatisation that can result from eczema; frustration and anxiety associated with the perceived lack of control and predictability of symptoms; and guilt for unknowingly contributing to a child's disease through life choices or family history.

**TABLE 1:** Top 10 topics collated from Natural Language Processing assessment of comments on Reddit, PatientInfo, BabyCenter, Talk Health Partnership, and Inspire

Topic	Comments	% Total
Psychosocial impacts	53 345	26.7
Moisturising	45 886	23
Itch	43 900	22
Steroid concerns	43 038	21.6
Triggers	39 354	19.7
Diet	34 015	17
Natural products	24 416	12.2
Dupixent	23 075	11.6
Flares	17 719	8.9
Bathing	17 214	8.6

*Note*: A post may include more than one topic and thus % total adds to more than 100%.

Additional parent/patient comments also were frequently related to moisturising regimen including questions about product selection or optimising of regimens as well as potential side effects of select skin care ingredients or products (Table 1). Consistent with prior analyses,^3^ complaints and comments about itch were the third most frequent topic followed by concerns about side effects of, and potential for withdrawal from, topical corticosteroids (Table 1).

Our results add to the literature suggesting substantial psychosocial burden and unmet mental health needs in patients who experience AD and their caregivers. They are consistent with prior survey data and meta‐analysis work identifying substantial psychosocial burdens; for example, survey data found that approximately 30% of patients with AD and 52% of AD caregivers report depression.^4^ A cohort study detected odds ratios for depression of 1.29–1.84 for mild‐moderate AD and 2.38 for severe AD.^5^ Findings of internalising behaviour were also more frequent in patients with AD, especially if their disease had an early age of onset.^5^ Additional publications linked AD to risk of suicide, stress, and overall dissatisfaction with life in both patients and caregivers.^6^, ^7^ Many medical conditions may unmask or exacerbate behavioural health conditions. However, chronic itch represents a special, underappreciated case wherein the biologic pathology of pruritis is related to the pathology of anxiety, beyond the psychosocial impacts of the disease. There is a positive feedback loop between anxiety and itch with evidence for neurologic correlates between the two.^8^ Further, the unpredictable nature of AD may further contribute to anxiety among patients who lack control over flares and symptoms.

Our study is limited by the inability to extract comments from social media sites with private messaging or non‐public posts. Furthermore, while over 250 000 posts were evaluated, we cannot yet evaluate how well this sample reflects an AD community that numbers in the tens of millions in the US alone. NLP techniques can be used to aggregate and sort topics but are less useful for determining the validity of information in posts. Therefore, while our analysis did not identify any specific false statements occurring with high frequency, given the volume of online discussions, both patients and providers may benefit from proactively addressing any unvetted medical information viewed online. Furthermore, while AD is by far the most common form of eczema, our NLP analysis cannot assure commentors were specifically referring to AD when discussing their ‘eczema’. However, NLP techniques such as topic modelling can uniquely assess candid descriptions of the issues facing patients and caregivers in their own words. NLP can also help pinpoint areas where clinical interventions and future research may ease their burdens. Our findings specifically suggest a need to expand both clinical care, community engagement, and research efforts surrounding the psychosocial impacts of AD.

## CONFLICT OF INTEREST

None to declare.

## FUNDING INFORMATION

Intramural Research Program of NIAID; NIH

## AUTHOR CONTRIBUTIONS

**Ying Yin:** Conceptualisation, Data curation, Formal analysis, Investigation, Project administration, Resources, Supervision, Visualization, Writing – original draft, Writing – review & editing; **Korey Capozza:** Conceptualisation, Data curation, Formal analysis, Investigation, Methodology, Project administration, Supervision, Validation, Visualization, Writing – original draft, Writing – review & editing; **Yijun Shao:** Conceptualisation, Data curation, Formal analysis, Investigation, Methodology, Resources, Software, Validation, Visualization, Writing – original draft; **Michelle Tu:** Data curation, Formal analysis, Investigation, Methodology, Software, Supervision, Validation, Writing – original draft; **Phillip Ma:** Conceptualisation, Data curation, Formal analysis, Funding acquisition, Investigation, Methodology, Project administration, Supervision, Writing – original draft; **Qing Zeng‐Treitler:** Conceptualisation, Data curation, Formal analysis, Investigation, Methodology, Project administration, Resources, Software, Supervision, Validation; **Ashleigh A Sun:** Data curation, Formal analysis, Resources, Software, Supervision, Writing – original draft; **Ian Antheni Myles:** Conceptualisation, Data curation, Formal analysis, Funding acquisition, Investigation, Project administration, Resources, Supervision, Visualization, Writing ‒ original draft, Writing ‒ review & editing, Methodology, Validation.

## Data Availability

Data sharing is not applicable to this article as no new data were created or analyzed in this study.
